# A subset of neutrophil phagosomes is characterised by pulses of Class I PI3K activity

**DOI:** 10.1242/dmm.052042

**Published:** 2025-08-29

**Authors:** Clare F. Muir, Constantino Carlos Reyes-Aldasoro, Tomasz K. Prajsnar, Bartosz J. Michno, Justyna Cholewa-Waclaw, Yin X. Ho, Audrey Bernut, Catherine A. Loynes, Stone Elworthy, Kieran A. Bowden, Ashley J. Cadby, Lynne R. Prince, Jason S. King, Felix Ellett, Alison M. Condliffe, Stephen A. Renshaw

**Affiliations:** ^1^ Bateson Centre, School of Medicine and Population Health, University of Sheffield, Sheffield S10 2TN, UK; ^2^ Royal (Dick) School of Veterinary Studies, University of Edinburgh, Edinburgh EH25 9RG, UK; ^3^ Centre of Inflammation, Institute for Regeneration and Repair, University of Edinburgh, Edinburgh EH16 4UU, UK; ^4^ Department of Computer Science, School of Science and Technology, City, University of London, London EC1V 0HB, UK; ^5^ Department of Evolutionary Immunology, Institute of Zoology and Biomedical Research, Faculty of Biology, Jagiellonian University, Kraków 30-387, Poland; ^6^ Doctoral School of Exact and Natural Sciences, Jagiellonian University, Kraków 30-348, Poland; ^7^ Laboratory of Pathogens and Host Immunity, Université de Montpellier, Montpellier 34090, France; ^8^ Department of Physics and Astronomy, University of Sheffield, Sheffield S3 7RH, UK; ^9^ School of Biosciences, University of Sheffield, Sheffield S10 2TN, UK; ^10^ Centre for Engineering in Medicine and Surgery, Department of Surgery, Massachusetts General Hospital, Harvard Medical School, Shriners Burns Hospital, Boston, MA 02114, USA

**Keywords:** Phagocytosis, Neutrophils, Zebrafish, Phosphatidylinositol (3,4,5)-trisphosphate, Phagosome maturation

## Abstract

Class I PI3 kinases (PI3Ks) coordinate the delivery of microbicidal effectors to the phagosome by forming phosphatidylinositol (3,4,5)-trisphosphate (PIP3). However, the dynamics of PIP3 in neutrophils during a live bacterial tissue infection are unknown. We therefore developed an *in vivo*, live zebrafish infection model that enables visualisation of dynamic changes in Class 1 PI3K signalling in neutrophil phagosomes in real time. We identified that, on ∼12% of neutrophil phagosomes, PHAkt-eGFP, a reporter for Class 1 PI3K signalling, repeatedly fades and re-recruits in pulsatile bursts. This phenomenon occurred on phagosomes containing live and dead bacteria as well as beads, and was dependent on the activity of the Class 1 PI3K isoform PI3Kγ. Detailed imaging suggested that pulsing phagosomes represent neutrophils transiently re-opening and re-closing phagosomes, a conclusion supported by observations that a subset of phagosomes in human neutrophils rapidly accumulate dye from the extracellular space. Therefore, we propose that some neutrophil phagosomes remain unsealed and are consequently able to exchange contents with the extracellular environment, with implications for phagosome fate and communication with surrounding cells.

## Teaser

**Summary:** Analyses in zebrafish and human neutrophils suggest that a subset of neutrophil phagosomes remain unsealed and are consequently able to exchange contents with the extracellular environment, with implications for phagosome fate and communication with surrounding cells.

## INTRODUCTION

Neutrophils are the most abundant white blood cells in the human body and are crucial in the immune system's early defence against bacterial infection. Neutrophils are avid phagocytes, capturing bacteria in membrane-bound vesicles called phagosomes for subsequent execution and digestion. The production of phosphatidylinositol (3,4,5)-trisphosphate (PIP3) on phagosome membranes by Class 1 PI3 kinases (PI3Ks) facilitates intraphagosomal killing of bacteria by coordinating reactive oxygen species (ROS) formation (Welch et al., 2005; Zhan et al., 2002), degranulation (Schreiber et al., 2010; Hoenderdos et al., 2016) and actin dynamics, enabling the phagosome to form and fuse with other organelles (Schlam et al., 2015; Bohdanowicz et al., 2010). However, although PIP3 has been shown to enable phagosome formation around large, inert prey [such as complement component 3 fragment (C3bi)-opsonised zymosan and IgG-opsonised erythrocytes] in neutrophil- and macrophage-like cells (Dewitt et al., 2006; Marshall et al., 2001), it is unknown how regulation of PIP3 in space and time controls the maturation of bacterial-containing phagosomes. It is also unknown which PI3K isoform generates PIP3 on neutrophil phagosomes. The pleckstrin homology domain of Akt (PHAkt) recognises PIP3 and also phosphatidylinositol (3,4)-bisphosphate [PI(3,4)P2; a degradation product of PIP3] and has been used extensively to characterise Class 1 PI3K activity in macrophage-like cell lines (Bohdanowicz et al., 2010; Marshall et al., 2001; Dormann et al., 2004; Giorgione and Clarke, 2008) and in neutrophil-like cell lines during migration (Servant et al., 2000) and phagocytosis (Dewitt et al., 2006). Under the control of the neutrophil-specific promoter, *mpx*, PHAkt has also been used to characterise the dynamics of PIP3 and PI(3,4)P2 in migrating zebrafish neutrophils (Yoo et al., 2010). By developing an *in vivo* infection model using another established transgenic zebrafish neutrophil reporter, *Tg(lyz:PHAkt-eGFP)i277* (Wang et al., 2014), we showed that PIP3/PI(3,4)P2 persists on neutrophil phagosomes far longer than *in vitro* macrophage models report (Bohdanowicz et al., 2010; Marshall et al., 2001; Dormann et al., 2004; Giorgione and Clarke, 2008) and that some PIP3/PI(3,4)P2-positive phagosomes repeatedly re-open and re-close, associated with re-recruitment of the reporter in a pulsatile manner. Pulsing occurs when both live and dead *Staphylococcus aureus* are ingested, as well as *Mycobacterium abscessus* and inert beads, and inhibitor studies suggest that this phenomenon is dependent on PI3Kγ. We propose that pulsing phagosomes are indicative of repeated cycles of phagosomes re-opening and re-closing and that this process follows incomplete sealing of the phagocytic cup.

## RESULTS

### PHAkt-eGFP recruits to neutrophil phagosomes

**Fig. 1.**
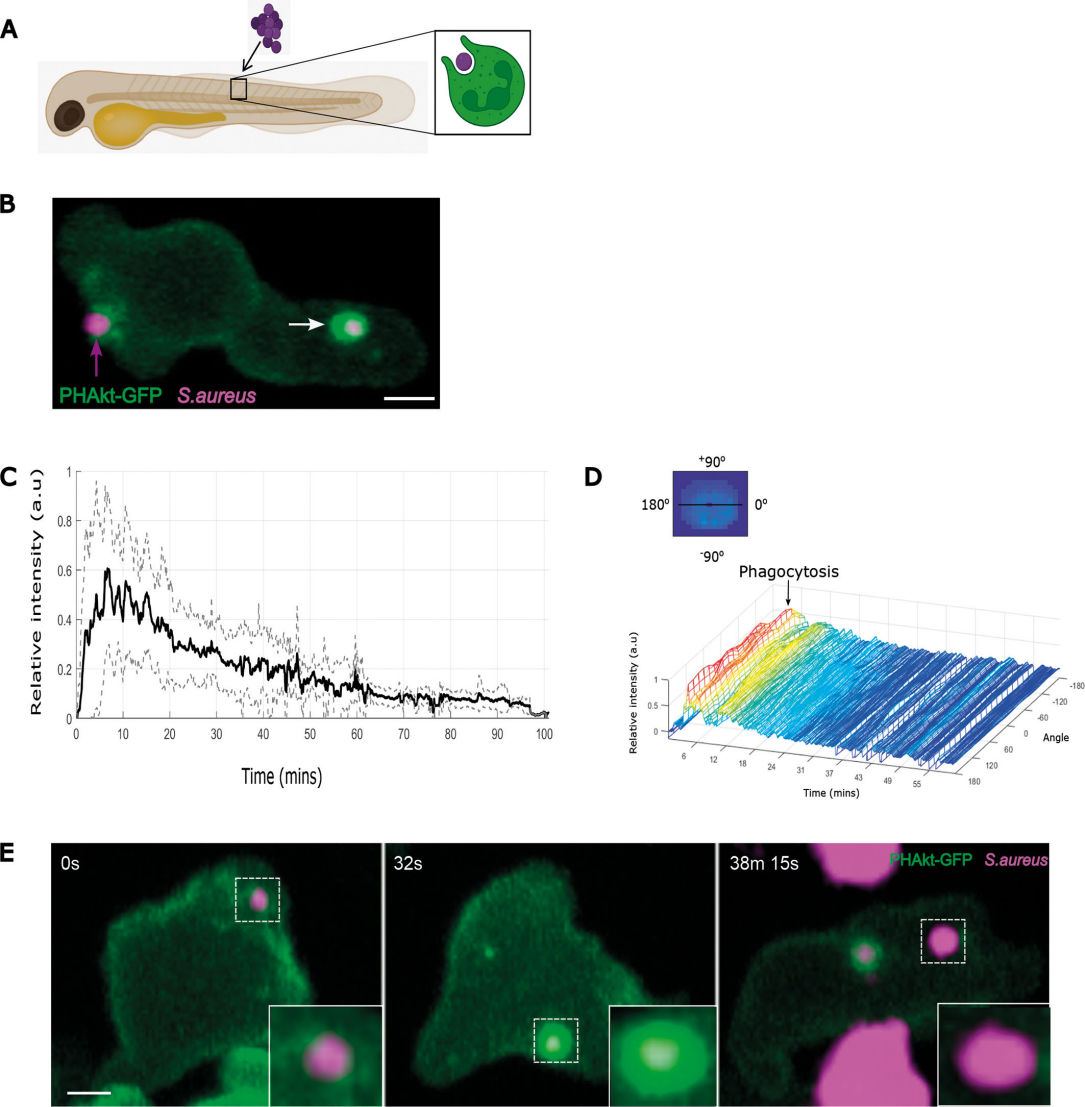
**Dynamics of PHAkt-eGFP on neutrophil phagosomes.** (A) Schematic illustrating localised somite injection of *S. aureus* into day 3 *Tg(lyz:PHAkt-EGFP)i277* zebrafish larvae. (B) Image from a timelapse capturing a neutrophil phagocytosing pHrodo™ Red-labelled *S. aureus* (magenta; magenta arrow) and strong recruitment of PHAkt-eGFP (green) to the phagosome after cup closure (white arrow). Scale bar: 2 µm. (C) Quantification of PHAkt-eGFP fluorescence on the phagosome membrane during phagocytosis of pHrodo™ Red-labelled *S. aureus*. Data shown are the average intensity values of nine phagosomes from four independent larvae, from four experiments±s.d. a.u., arbitrary units. (D) Quantification of PHAkt-eGFP fluorescence on the phagosome membrane over time. Graphic illustrates how intensity values around the phagosome membrane ('angle') were measured. (E) Sequential images illustrating PHAkt-eGFP recruitment to a neutrophil phagosome. Large clusters of strongly acidified bacteria are present within adjacent unlabelled, presumed macrophages. Scale bar: 2 µm.

To investigate the role of PIP3/PI(3,4)P2 during phagocytosis, we injected *S. aureus*, an important pathogen of humans and animals, locally into the somite of the zebrafish neutrophil PIP3/PI(3,4)P2 reporter line, *Tg(lyz:PHAkt-eGFP)i277* (Wang et al., 2014) (Fig. 1A). Minimal PHAkt-eGFP recruitment was detected during early formation of the phagocytic cup. However, as the phagocytic cup closed, PHAkt-eGFP localised distinctly to sites of cup closure and then, following cup closure, PHAkt-eGFP recruited strongly and uniformly to the entire phagosome membrane (Fig. 1B; Movie 1). Following the surge in PHAkt-eGFP recruitment, thin PHAkt-eGFP-positive tubules extended into the cytoplasm from all phagosomes, and areas of most intense reporter localisation appeared where PHAkt-eGFP-positive tubules recoiled back to join the phagosome membrane. PHAkt-eGFP then gradually diminished in the majority of phagosomes (Fig. 1C-E). However, we observed – to our surprise – that, in a subset of phagosomes, PHAkt-eGFP re-recruited to the phagosome membrane in pulsatile bursts (Movie 2), a phenomenon we then investigated in more detail.

### PHAkt-eGFP recruits in pulsatile bursts to neutrophil phagosomes

**Fig. 2.**
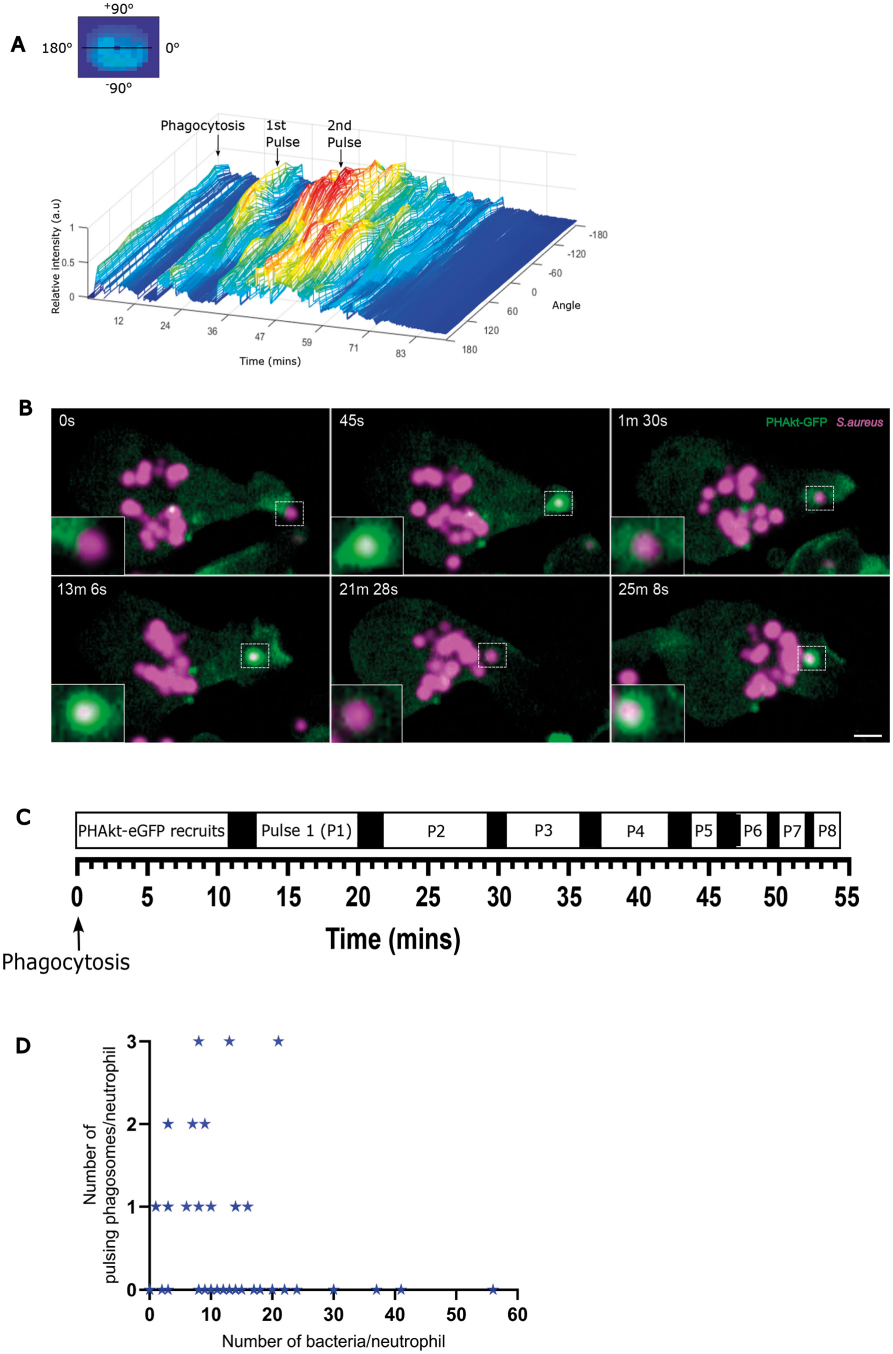
**PHAkt-eGFP recruits in pulsatile bursts to neutrophil phagosomes.** (A) Quantification of PHAkt-eGFP fluorescence on a pulsing phagosome. Graphic illustrates how intensity values around the phagosome membrane (‘angle’) were measured. (B) Sequential images of a pulsing phagosome. 0 s, start of phagocytosis; 45 s, surge of PHAkt-eGFP recruitment as the phagosome closes; 1 min 30 s, PHAkt-eGFP diminishes from the phagosome; 13 min 6 s, 1st pulse; 21 min 28 s, loss of PHAkt-eGFP recruitment; 25 min 8 s, 2nd pulse. Scale bar: 2 µm. (C) Schematic illustrating pulsatile recruitment of PHAkt-eGFP to phagosomes. Data represent average values from 152 phagosomes, from 11 independent larvae, from 11 experiments. (D) Pulses occur irrespective of the number of bacteria within a neutrophil. Data shown are from 55 neutrophils, from 11 independent larvae, from 11 experiments. Spearman's rank correlation between the number of pulsing phagosomes per neutrophil and the number of bacteria per neutrophil, was −0.2733; *P*=0.0415.

A pulse was defined as a transient but intense surge in PHAkt-eGFP recruitment to the phagosome (Fig. 2A,B; Movie 2). Repeated pulsing occurred on 12.4±24.9% (mean±s.d.) of phagosomes within a neutrophil, and 35.8±36.7%) of all phagocytic neutrophils had at least one pulsing phagosome. Cycles of PHAkt-eGFP diminishment and then re-recruitment usually started within 10.5±12.49 min of phagocytosis, and re-recruitment often occurred multiple times (Fig. 2C), typically four times on each phagosome, but with up to 18 pulses observed on one phagosome. Additionally, we identified that, although 17% of neutrophil phagosomes fused to form larger phagosomes, pulsing phagosomes never fused with other phagosomes and always contained only one bacterium. Some neutrophils phagocytosed more bacteria than others, and we wondered whether these hungry phagocytes had more pulsing phagosomes, perhaps reflecting exhaustion of phagocytic capacity (Hellebrekers et al., 2017). However, we observed the opposite. Pulsing phagosomes occurred more often in less phagocytic neutrophils, with no pulsing phagosomes observed in neutrophils that contained more than 22 bacteria (Spearman's rank correlation=−0.2733, *P*=0.0415) (Fig. 2D).

### Pulsatile bursts of PHAkt-eGFP recruitment are neutrophil initiated

**Fig. 3.**
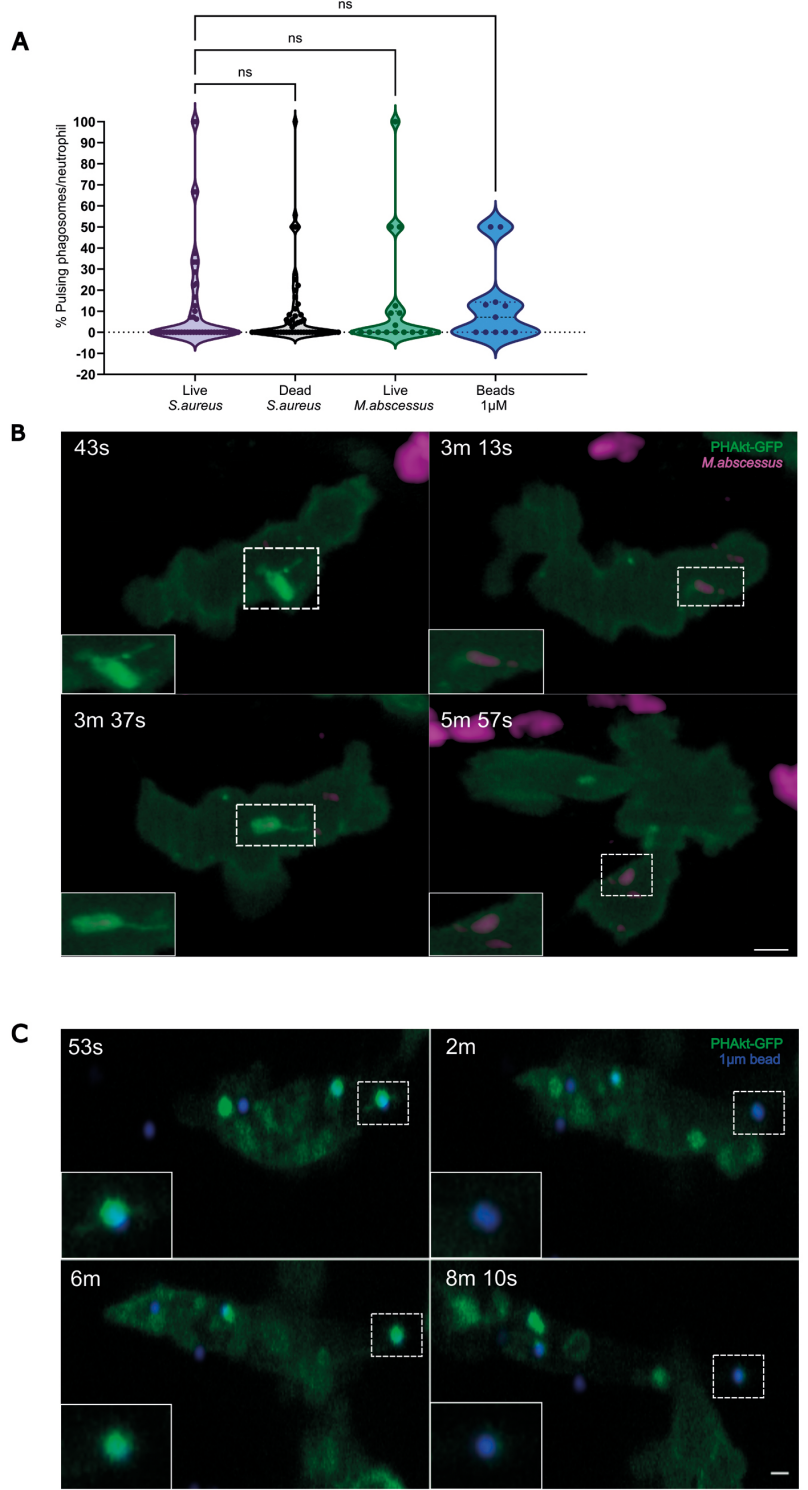
**Pulsatile bursts of PHAkt-eGFP recruitment are a neutrophil response to prey.** (A) Violin plot showing the percentage of pulsing phagosomes per phagocytic neutrophil for live and dead *S. aureus*, live *M. abscessus* and 1 µm beads. Data shown are the median with the 25th and 75th percentiles. Live *S. aureus*, 46 phagosomes analysed from 11 independent larvae, from 11 experiments; dead *S. aureus*, 54 phagosomes, ten independent larvae, from ten experiments; live *M. abscessus*, 18 phagosomes, six independent larvae, six experiments; 1 µm beads, 11 phagosomes, four independent larvae, four experiments. ns, not significant (Kruskal–Wallis test). (B) ‘Pulses’ occur on phagosomes containing *M. abscessus*. PHAkt-eGFP recruits to phagosomes during phagocytosis (43 s) before gradually diminishing (3 min 13 s). PHAkt-eGFP then re-recruits to the phagosome (1st pulse; 3 min 37 s) and then diminishes again (5 min 57 s). Scale bar: 2 µm. (C) Pulses occur on phagosomes containing 1 µm beads. PHAkt-eGFP recruits to phagosomes during phagocytosis (53 s) before gradually diminishing (2 min). PHAkt-eGFP then re-recruits to the phagosome (1st pulse; 6 min) and then diminishes again (8 min 10 s). Phagocytosis starts at 0 min. Scale bar: 1 µm.

Having observed that PHAkt-eGFP re-recruits to phagosomes containing live *S. aureus*, we wondered whether this phenomenon reflected bacterial manipulation of PIP3/PI(3,4)P2 signalling. To investigate this, *Tg(lyz:PHAkt-eGFP)i277* larvae were injected with heat-killed *S. aureus* (Fig. 3A), hypothesising that pulsing would not be observed if it was due to an active bacterial response to neutrophil attack. However, no significant difference was identified between the percentage of pulsing phagosomes in neutrophils containing live (12.4±24.9%) versus dead (9.3±19.0%) bacteria. Pulsing therefore, does not appear to reflect active manipulation of PIP3/PI(3,4)P2 signalling by *S. aureus*.

To investigate whether phagosomal ‘pulsing’ is a specific response to *S. aureus*, larvae were injected with a structurally and morphologically distinct prey, *M. abscessus* (Fig. 3A,B; Movie 3). Pulsatile recruitment of PHAkt-eGFP was again identified, and the frequency of pulses was similar to that in phagosomes containing *S. aureus* (13±26.9%) (Fig. 3A), indicating that PHAkt-eGFP re-recruitment occurs irrespective of the species of bacteria engulfed. To distinguish whether pulsing is a neutrophil response to phagocytosis of bacteria versus non-bacterial prey, larvae were injected with 1 µm polystyrene beads (F13083; a size similar to *S. aureus*) (Fig. 3C; Movie 4). Again, a similar frequency of pulsing was identified (13.36±19.02%) (Fig. 3A). Together, these data suggest that pulsing is a normal neutrophil response to internalisation of potential prey, rather than bacteria attempting to manipulate host signalling.

### pH change in pulsing phagosomes differs from that in non-pulsing phagosomes

**Fig. 4.**
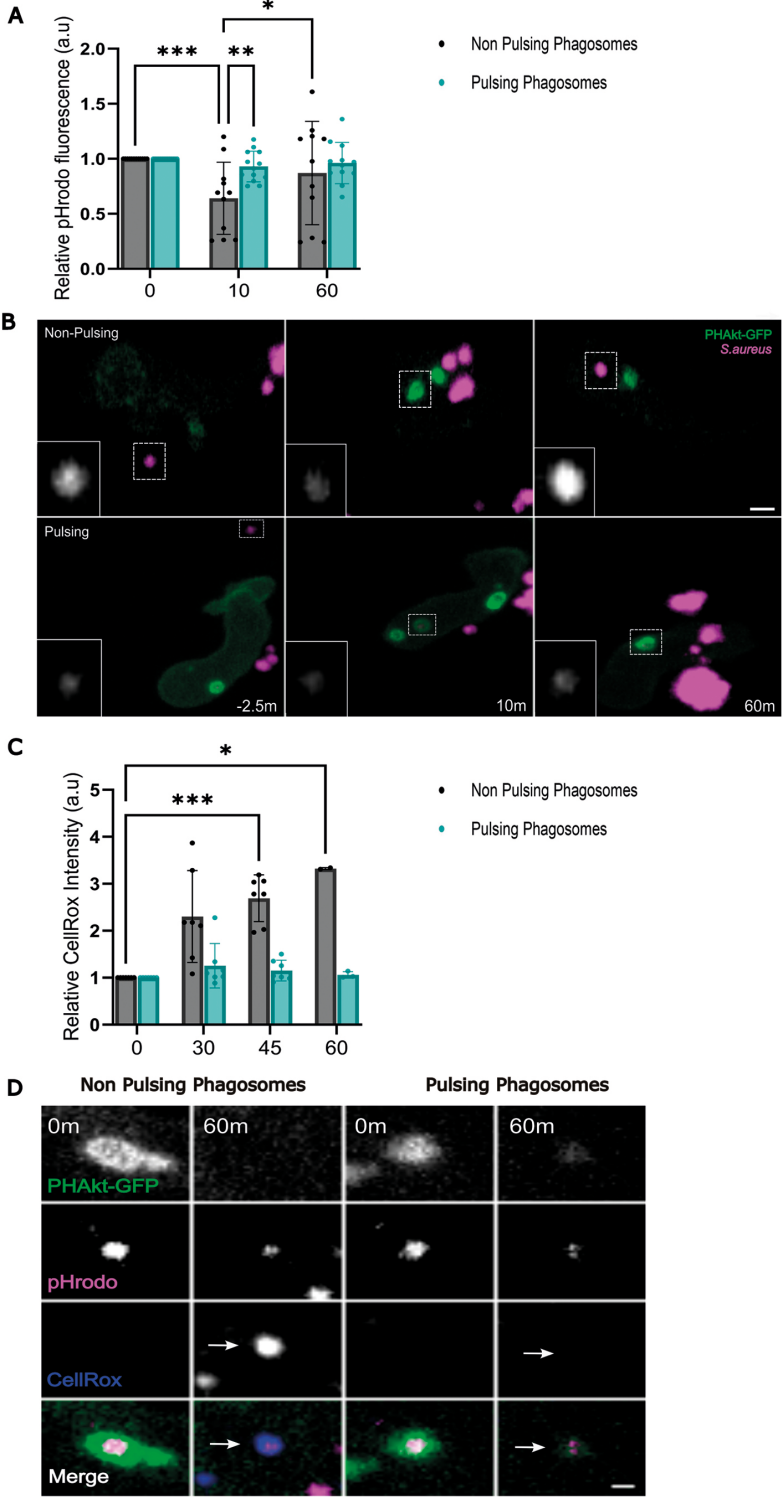
**pH change in pulsing phagosomes differs from that in non-pulsing phagosomes.** (A) Quantification of the relative change in fluorescence of pHrodo-stained *S. aureus* in non-pulsing versus pulsing phagosomes. Data shown are the mean±s.d. 0 min, 12 non-pulsing phagosomes, 14 pulsing phagosomes; 10 min, 11 non-pulsing phagosomes, 13 pulsing phagosomes; 60 min, 11 non-pulsing phagosomes, 12 pulsing phagosomes, 11 independent larvae, 11 experiments. (B) Sequential timelapse images comparing the change in fluorescence of pHrodo-stained *S. aureus* in pulsing versus non-pulsing phagosomes. Scale bars: 2 µm. (C) Quantification of the relative change in CellROX™ fluorescence within non-pulsing versus pulsing phagosomes. Data shown are the mean±s.d. 0 min, seven non-pulsing phagosomes, seven pulsing phagosomes; 30 min, seven non-pulsing phagosomes, seven pulsing phagosomes; 45 min, seven non-pulsing phagosomes, seven pulsing phagosomes; 60 min, two non-pulsing phagosomes, three pulsing phagosomes; from four independent larvae, from four experiments. (D) Sequential images illustrating changes in CellROX™ fluorescence within non-pulsing versus pulsing phagosomes. Arrows indicate bright fluorescence of CellROX in a non-pulsing phagosome vs absence of fluorescent CellROX within a pulsing phagosome, 60 min after phagocytosis. Scale bar: 1 µm. *P<0.05, **P<0.005, ***P<0.001 (mixed-effects model with multiple comparisons).

We then wished to establish whether pulsing phagosomes mature differently to non-pulsing phagosomes and whether they have the same bactericidal capacity. The pH of human neutrophil phagosomes rises to pH 8.5-9.0 immediately after particle engulfment, and this elevated pH is maintained for 20-30 min (Levine et al., 2015; Foote et al., 2019, 2017). The rise in pH is attributed to the activity of NADPH oxidase as NADPH inhibition minimises alkalinisation (Fig. S1A-C). NADPH oxidase regulates phagosomal pH through several mechanisms, including consumption of H^+^ during H_2_O_2_ production (Segal et al., 1981), reducing V-ATPase insertion into the membrane (Jankowski et al., 2002) and increasing H^+^ leakage through conductive channels (Jankowski et al., 2002). Limited studies have examined how the neutrophil phagosome changes 30 min after phagocytosis. However, studies using fluorescein-stained particles have demonstrated that the neutrophil phagosome gradually acidifies to pH 5.6/6 after 60-120 min (Segal et al., 1981; Cech and Lehrer, 1984); however, these studies may not be accurate as fluorescein fluorescence dims following exposure to ROS (Platkov et al., 2014). pHrodo™ fluorescence increases in acidic environments but can still be visualised at pH 9 (Fig. S1D) (Dolman et al., 2013). pHrodo™ staining also does not affect bacterial viability and pathogenicity in the zebrafish infection model (Fig. S2A). Therefore, we choose pHrodo™ as a read-out to establish whether the pH of pulsing versus non-pulsing phagosomes differs. In non-pulsing phagosomes, the fluorescence of pHrodo™-stained *S. aureus* was significantly dimmer 10 min after phagocytosis before returning to a similar brightness to that pre-phagocytosis (Fig. 4A,B). In contrast, within pulsing phagosomes, the fluorescence of pHrodo™-stained *S. aureus* remained similar to that pre-phagocytosis at 10 min and 60 min post phagocytosis (Fig. 4A,B). As pHrodo™ fluorescence dims in alkaline pH (Fig. S1D) (Wood et al., 2020; Prajsnar et al., 2021), these findings suggest that pulsing phagosomes alkalinise less than non-pulsing phagosomes shortly after phagocytosis. Additionally, within pulsing phagosomes, there was no significant change in CellROX™ fluorescence (Fig. 4C,D). However, within non-pulsing phagosomes, the fluorescence of CellROX™ significantly increased 45 min post phagocytosis (Fig. 4C,D). This finding suggests that there is diminished ROS production in pulsing phagosomes and/or that pulsing phagosomes fail to accumulate CellROX™ dye. One potential explanation is that pulsing phagosomes remain open to the external environment.

### Pulsatile recruitment of PHAkt-eGFP identifies phagosomes that repeatedly re-open and re-close

**Fig. 5.**
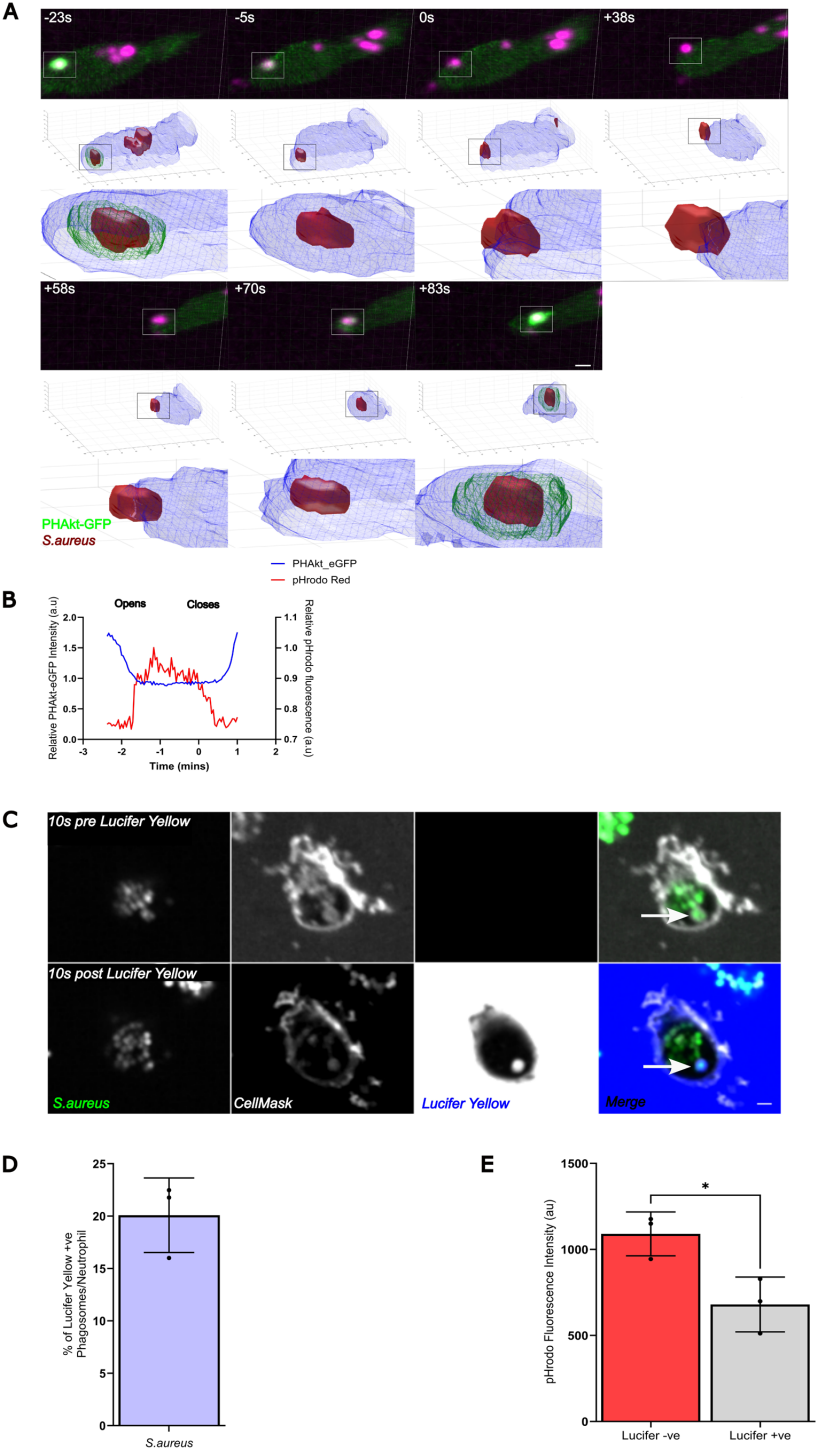
**A subset of neutrophil phagosomes may remain unsealed.** (A) Sequential images and 3D reconstruction of PHAkt-eGFP dynamics when *S. aureus* is within a phagosome (−23 s), the phagosome re-opens (0 s) and then re-closes (+70 s). Scale bar: 2 µm. (B) Quantification of the change in PHAkt-eGFP fluorescence of pHrodo-stained *S. aureus* when a neutrophil phagosome re-opens and closes. (C) Images illustrating the accumulation of Lucifer Yellow in a formed human neutrophil phagosome (arrows). There was a 20 s delay between imaging, adding Lucifer Yellow and then re-commencing imaging as we needed to unload the plate from the microscope to add the dye. Scale bar: 1 µm. (D) Quantification of the percentage of Lucifer Yellow^+^ phagosomes in human neutrophils at 30 months post infection (mpi). Data shown are the mean±s.d. of 2779 phagosomes analysed from three experiments. (E) Quantification of pHrodo fluorescence in Lucifer Yellow^−^ versus Lucifer Yellow^+^ phagosomes at 60 mpi. Data shown are the mean±s.d. of 4369 phagosomes analysed from three experiments. *P<0.05 (unpaired *t*-test).

During our experiments, we observed neutrophils expelling *S. aureus* from PHAkt-eGFP-positive phagosomes (Movie 5). Neutrophils often re-phagocytosed the same bacteria, and, in this setting, PHAkt-eGFP once again recruited to the enveloping membranes. During the surge in PHAkt-eGFP recruitment, we also identified that the neck of 9.7% (3/31) of phagosomes during a pulsing event formed an elongate tubule, remaining connected to the plasma membrane. Therefore, we considered it possible that such phagosomes had not closed fully or that closure might be imperfect/incomplete. Imaging of this phenomenon was challenging as phagosomes often only opened transiently and via a small pore. However, 3D reconstruction of phagosomes (Fig. 5A; Movie 6) enabled us to visualise re-opening of 19.6% (27/138) of phagosomes during a pulse. On re-opening, PHAkt-eGFP recruitment rapidly dropped, with the bacteria usually remaining within the newly opened phagocytic cup, before the phagosome resealed (Fig. 5A; Movie 6). To confirm that pulsing phagosomes re-open, actin and PHAkt dynamics were assessed in parallel using *Tg(lyz:PHAkt:GFP)i277*×*Tg(mpx:Lifeact-Ruby)SH608* larvae. This showed that cortical actin filaments separated, as pulsing phagosomes approached the neutrophil surface and remained on either side of the phagosome as the phagocytic cup opened to release the prey. After the phagocytic cup re-formed and re-captured prey, cortical actin filaments then propelled the phagosome inwards once again (Fig. S3A). This confirmed that PHAkt-eGFP decreases when phagosomes re-open and release bacteria, and that PHAkt re-recruits to phagosomes when they re-capture expelled bacteria (pulsing). We also observed a distinct increase in the fluorescence of pHrodo™-stained *S. aureus* when bacteria were released into the tissue, a less alkaline environment than the early neutrophil phagosome (Foote et al., 2019; Jankowski et al., 2002) (Fig. 5B). The fluorescence of pHrodo™-stained *S. aureus* then decreased as the bacteria were re-phagocytosed, as is expected as neutrophil phagosomes rapidly alkalinise during phagocytosis (Foote et al., 2019; Pazhakh et al., 2019) (Fig. 5B). In 5.1% (7/138) of ‘pulses’, bacteria were fully released from the phagocytic cup but remained close to the neutrophil surface before being re-captured. We observed that 94% (29/31) of bacteria from pulsing phagosomes were successfully re-captured by the same neutrophil, 3% (1/31) of bacteria were released and then re-phagocytosed by a non-fluorescent phagocytic cell (presumed macrophage), and 3% (1/31) of bacteria were shuttled into another neutrophil (Greene et al., 2022).

Having observed that some pulsing phagosomes re-open, we wished to determine whether this phenomenon followed failure of the phagosome to seal or whether this followed an active exocytosis event. To achieve this, we allowed human neutrophils to phagocytose *S. aureus* for 30 min and then added the cell membrane-impermeable dye Lucifer Yellow. We observed that 20.1±2.9% of phagosomes containing live *S. aureus* rapidly accumulated Lucifer Yellow fluorescence (Fig. 5C,D). Given that simultaneous exocytosis events would be unlikely to occur for this number of phagosomes (Hawkins and Stephens, 2015), we propose that these leaky phagosomes remain unsealed. Additionally, the fluorescence of pHrodo™-stained *S. aureus* was less bright within phagosomes that accumulated Lucifer Yellow (Fig. 5E), suggesting that unsealed phagosomes acidify less than those that remain sealed.

### Pulses of PHAkt-eGFP are abolished by PI3Kγ inhibition

**Fig. 6.**
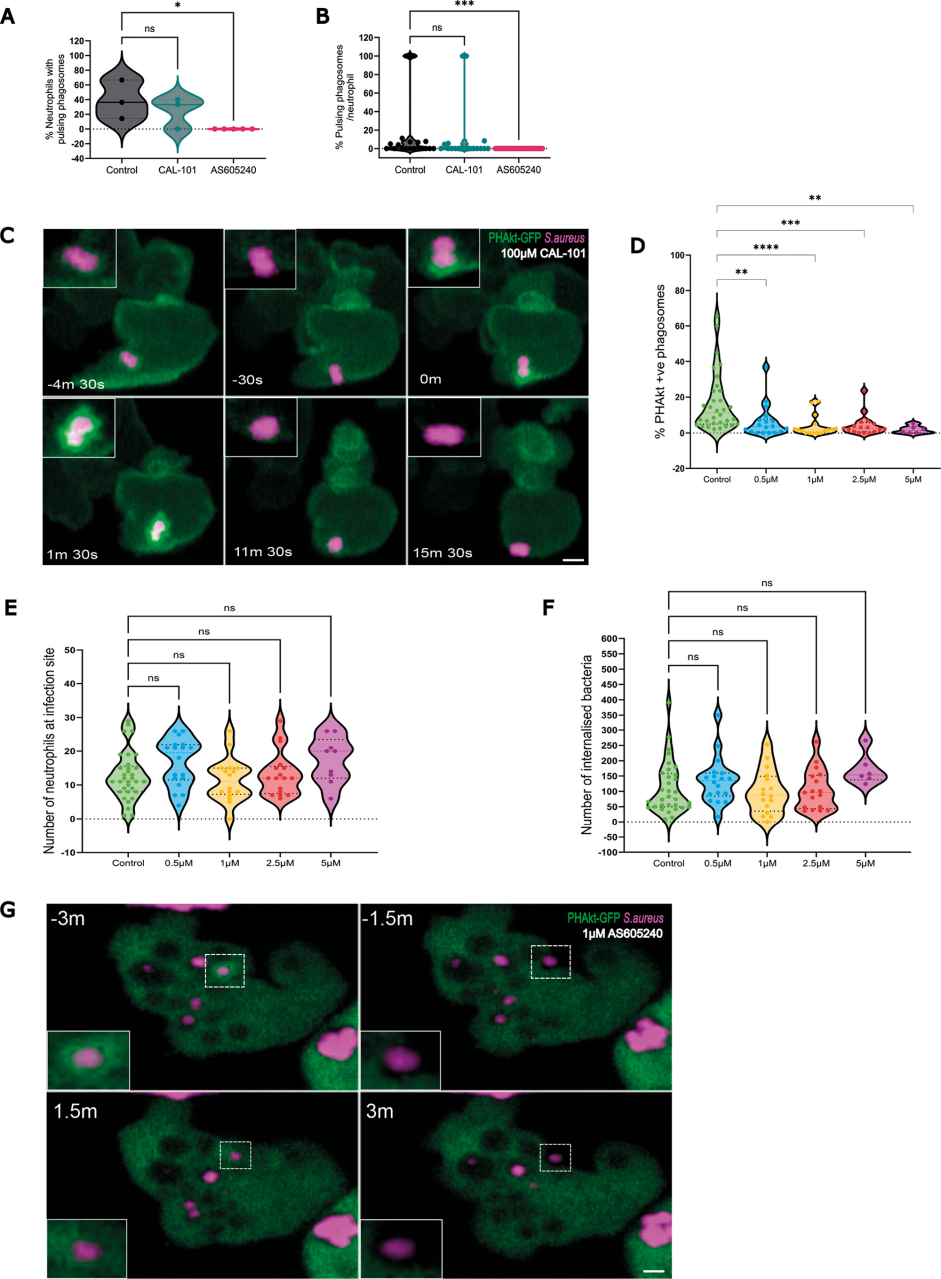
**Pulses of PHAkt occur despite inhibition with CAL-101 and are abolished by AS605240.** (A) Violin plot showing the percentage of neutrophils that have pulsing phagosomes. Timelapse started 2 h post infection following 30 min incubation of *Tg(lyz:PHAkt-EGFP)i277* larvae with 100 µm CAL-101 and 1 µm AS605240. Data shown are the median with the 25th and 75th percentiles from 87 neutrophils, 11 independent larvae, 11 experiments. (B) Violin plot showing the percentage of pulsing phagosomes in each neutrophil in a timelapse starting 2 h post infection following 30 min incubation of *Tg(lyz:PHAkt-eGFP)i277* larvae with 100 µm CAL-101 and 1 µm AS605240. Data shown are the median with the 25th and 75th percentiles from 780 phagosomes, 11 independent larvae, 11 experiments. (C) Sequential images illustrating pulsatile recruitment of PHAkt-eGFP to a phagosome exposed to 100 µm CAL-101. Scale bar: 2 µm. (D) Violin plot showing quantification of the percentage of PHAkt-eGFP^+^ phagosomes 2 h post infection following 30 min incubation with AS605240. Data shown are the median with the 25th and 75th percentiles from six independent larvae, from six experiments. (E) Violin plot showing quantification of the number of neutrophils at a *S. aureus* infection site 2 h post infection following 30 min incubation with AS605240. Data shown are the median with the 25th and 75th percentiles from six independent larvae, six experiments. (F) Violin plot showing quantification of the number of bacteria/neutrophils 2 h post infection following 30 min incubation with AS605240. Data shown are the median with the 25th and 75th percentiles from six independent larvae, six experiments. (G) Sequential images illustrating that PHAkt-eGFP recruits to neutrophil phagosomes prior to exposure to 1 µm AS605240 (3 min before attempted re-phagocytosis (−3 min). Bacteria are then released from the phagosome (−1.5 min). Following exposure to 1 µm AS605240, the neutrophil attempts to re-phagocytose the bacteria, but PHAkt-eGFP does not recruit to the phagosome (1.5 min), and the bacteria are released from the phagosome (3 min). Scale bar: 2 µm. *P<0.05, **P<0.005, ***P<0.001, ****P<0.0001 (Kruskal–Wallis with multiple comparisons).

During imaging, we identified that PHAkt-eGFP uniformly increases on the phagosome membrane as the phagocytic cup closes and that there is a second increase in PHAkt-eGFP recruitment at the neck of the phagosome, prior to sealing. We next aimed to identify the Class 1 PI3K enzyme that drives dynamic PIP3 production on neutrophil phagosomes (Sadhu et al., 2003). The three tyrosine kinase-linked Class 1A PI3K isoforms are PI3Kα, PI3Kβ and PI3Kδ, and the sole G-protein activated Class 1B PI3K isoform is PI3Kγ (Sadhu et al., 2003). Neutrophils express abundant PI3Kγ and PI3Kδ (Sadhu et al., 2003), and, in zebrafish, inhibition of PI3Kγ (also known as PIKCG) completely abolishes the migration of neutrophils to an injured tail fin (Yoo et al., 2010). Although unexplored in a zebrafish infection model, PI3Kδ inhibition has been reported to reduce the migration of human neutrophils *in vitro* (Elworthy et al., 2023) and also reduces neutrophil migration towards a TNFα injection site in mouse. This makes it difficult to assess whether neutrophils from transgenic fish lacking PI3Kγ or PI3Kδ (also known as PIK3CD) phagocytose prey *in vivo*, as such neutrophils will not migrate to the infection site. To assess the role of PI3Kγ and PI3Kδ in phagocytosis, neutrophils were therefore allowed to migrate to the infection site for 60 min, and then zebrafish were exposed to class 1 PI3K inhibitors. Based on our previous work using these inhibitors in zebrafish models (Lannutti et al., 2011), we incubated larvae for 30 min with 100 µm of the PI3Kδ inhibitor, CAL-101 [half-maximal inhibitory concentration (IC50)=2.5 nM; half-maximal effective concentration (EC50)=8 nM (Camps et al., 2005)] before immediately commencing imaging. The relatively high concentration of the inhibitor reflects the need for penetration into the tissues. Although CAL-101 decreased both the total number of neutrophils and the number of phagocytic neutrophils at an infection site (Fig. S4A,B), in both control zebrafish and zebrafish exposed to CAL-101, pulsatile bursts of PHAkt-eGFP recruitment occurred on neutrophil phagosomes (Fig. 6A-C). These data suggest that PI3Kδ does not mediate the pulsatile recruitment of PHAkt-eGFP to neutrophil phagosomes.

We titrated the dose of PI3Kγ inhibitor AS605240 [IC50=8 nM (Williams et al., 2010); EC50=117 nM (Gilbert et al., 2017)] to identify that a delayed 30 min incubation with 1 µm of this compound significantly decreased the percentage of PHAkt-eGFP-positive phagosomes, but not the number of neutrophils recruited to the infection site or the number of bacteria internalised per neutrophil (Fig. 6D-F). This suggested to us that neutrophils recruited to the infection site and phagocytosed bacteria in the 60 min before the PI3Kγ inhibitor was applied. However, the subsequent application of AS605240 then reduced the production of PIP3/PI(3,4)P2 on neutrophil phagosomes (Fig. 6G; Movie 7). Close examination of timelapses of AS605240-treated larvae showed that although some PHAkt-eGFP recruited to neutrophils as they initiated phagocytosis, the later larger surges of PHAkt-eGFP recruitment, first to the fully formed phagocytic cup and second to the neck of the phagosome during closure, did not occur. In the absence of PHAkt-eGFP recruitment to phagosomes, the partially phagocytosed bacteria were released back into the tissue. Occasionally, neutrophils attempted to re-phagocytose the bacteria, but, again, visible re-recruitment of PHAkt-eGFP (pulsing) was absent (Fig. 6A,B,G; Movie 7). We, therefore, concluded that PI3Kγ enables phagocytosis. We propose that, without functional PI3Kγ activity, initiated neutrophil phagosomes fail to close effectively, and prey is subsequently released into the tissue but not re-captured.

## DISCUSSION

Using an *in vivo* model of Class 1 PI3K signalling, we have identified an intriguing new phenomenon by which neutrophils repeatedly re-open and re-close a significant proportion of phagosomes. This phenomenon occurs following phagocytosis of structurally and morphologically distinct bacteria, as well as inert beads.

In the majority of phagosomes, we were unable to visualise continuity between the phagosome and cell membrane using *in vivo* imaging techniques. It is therefore possible that pulsing phagosomes have fully sealed and then re-fused with the plasma membrane to release prey (non-lytic exocytosis) (Johnston and May, 2010; Bain et al., 2012; Quinn et al., 2021). However, using human neutrophils, we demonstrate that a subset of formed neutrophil phagosomes rapidly accumulate dye from the extracellular space. Given that simultaneous exocytosis events would be unlikely to occur for this number of phagosomes (Hawkins and Stephens, 2015), we suggest that these ‘leaky’ phagosomes remain connected to the extracellular space. Unsealed phagosomes may repeatedly re-open and close and thus generate pulses of Class 1 PI3K activity as the neutrophil attempts to re-capture partially released bacteria.

We speculate that unsealed phagosomes aid the release of partially degraded material into the extracellular space and that this facilitates exchange between the phagosome and the infected tissue. A process, termed ‘eructophagy’, occurs in primary human and murine macrophages (Hawkins and Stephens, 2015) and describes the release of partially digested bacterial DNA into the extracellular space that then activates pro-inflammatory signalling pathways in neighbouring cells (Hawkins and Stephens, 2015). Eructophagy occurs in ∼10% of phagolysosomes over a 3 h period and is stimulated by inflammation and nutrient depletion. However, eructophagy only occurs 2-10 h after phagocytosis and in phagosomes that have acidified and fused with lysosomes (Hawkins and Stephens, 2015) (i.e. after phagosome maturation). In contrast, we propose that, shortly after phagocytosis, a minority of nascent neutrophil phagosomes remain unsealed and connected to the extracellular environment.

We identified that recruitment of PHAkt to neutrophil phagosomes is dependent at least in part on PI3Kγ activity. This suggests that PI3Kγ, in addition to playing a key role in enabling neutrophil migration (Servant et al., 2000), enables bacteria-containing phagosomes to seal. Supporting our findings, Quinn et al. (2021) identified that Class 1 PI3Ks, which include PI3Kγ, enable macropinosomes to seal (Deladeriere et al., 2015), and potentially similar mechanisms may underpin phagocytosis of bacteria. There are two regulatory subunits for PI3Kγ – namely, p84 and p101 – each of which coordinates different neutrophil responses (Suire et al., 2006; Rathinaswamy et al., 2021). Future work may determine which PI3Kγ regulator(s) enable PIP3 production on bacterial-containing phagosomes to facilitate closure. This is important as it may allow selective therapeutic targeting of neutrophil function. For example, although there are currently no p84/p101 inhibitors, selective nanobodies have recently been developed that can selectively target the different PI3Kγ inhibitory complexes (Fey et al., 2013).

Additionally, in contrast to neutrophils phagocytosing yeast *in vitro* (Dewitt et al., 2006), the intensity of PHAkt-eGFP only slightly increased as neutrophil membranes wrapped around bacteria. Instead, PHAkt-eGFP intensity increased, first, as opposing membranes appeared to touch and, second, with greatest intensity, on the entire phagosome as the phagosome appeared to be internalised. This may reflect differences in how PI3Ks coordinate phagocytosis in neutrophils versus macrophages, zebrafish versus human cells and/or the phagocytic target, e.g. Ig-opsonised beads versus bacteria.

In conclusion, we have developed an *in vivo* model to image Class1 PI3K signalling in real time during a bacterial infection. This model has enabled us to identify that PI3Kγ produces the majority of PIP3/PI(3,4)P2 on bacterial phagosomes and that the majority of PI3Kγ activity occurs after bacteria are internalised by the neutrophil. Additionally, pulses of class 1 PI3K activity occur when neutrophils repeatedly re-open and re-close phagosomes, and a slightly higher proportion of human neutrophil phagosomes rapidly accumulate dye when it is added to the extracellular space. Our quantification of the percentage of unsealed phagosomes in zebrafish may be an underestimate as we could only identify phagosome re-opening by a pulse of Class 1 PI3K activity, rather than the more accurate accumulation of dye from the extracellular space. This intriguing finding suggests that a subset of neutrophil phagosomes remain open, and we speculate that this may be a normal part of neutrophil phagosome maturation to aid exchange between the intracellular and extracellular environment.

## MATERIALS AND METHODS

### Experimental model details

Animal work was carried out according to guidelines and legislation set out in UK law in the Animals (Scientific Procedures) Act 1986, under Project License PP7684817. Ethical approval was granted by the University of Sheffield Local Ethical Review Panel. Adult zebrafish and larvae were maintained in the Bateson Centre aquarium at the University of Sheffield and maintained according to standard protocols (Prajsnar et al., 2012). The aquarium is a continuous re-circulating closed system with a light/dark cycle of 14/10 h, respectively, and a temperature of 28°C. Existing lines were London wild type (LWT), Nacre and *Tg(lyz:PHAkt-eGFP)i277*. Larvae were maintained in E3 medium (5 mM NaCl, 0.17 mM KCl, 0.33 mM CaCl_2_, 0.33 mM MgSO_4_) plus Methylene Blue (Sigma-Aldrich, 50,484) at 28°C until 3 days post fertilisation.

### Method details

#### Bacterial strain and growth conditions

*S. aureus* strain USA 300 (Bernut et al., 2014) and *M. abscessus* strain CIP104536T morphotype smooth were used for all experiments (Kwan et al., 2007). Overnight cultures of *S. aureus* were started from single colonies and grown in 10 ml brain heart infusion medium (Sigma-Aldrich, 53286) overnight at 37°C, shaking at 250 rpm. In the morning, cultures were diluted 1:100 and grown for 2.5 h. Cultures were pelleted at 4500 ***g*** for 15 min at 4°C (Sigma-Aldrich, 3-16KL, 11,240×13,145 rotor) and re-suspended in PBS (Oxoid, BR0014 G) to 1500 colony-forming units (cfu)/nl. For specific experiments, *S. aureus* were killed by heating in an 80°C water bath for 20 min. *M. abscessus* cultures were grown and prepared for infection challenges (150 cfu/nl) as previously described (Kwan et al., 2007).

#### pHrodo™ staining of bacteria

200 µl of PBS/bacterial suspension was added to 1 µl pHrodo™ Red (Thermo Fisher Scientific, P36600 or P35369), incubated at 37°C, 100 rpm for 30 min. Stained bacteria were then washed with 1 ml PBS and 1 ml 30 mM Tris-HCl pH 8.5, before a final wash with 1 ml PBS and resuspension in PBS for injection.

#### Zebrafish injections

Zebrafish larvae were injected using borosilicate glass needles (World Precision, Instruments, TW100-4) and a pneumatic PicoPump (World Precision Instruments, PV820). Borosilicate glass needles were prepared using a horizontal micropipette puller (Sutter Instruments, P-1000 Flaming/Brown™) and subsequently loaded with 7 μl inoculum prepared as above using an Eppendorf™ Microloader™ Pipette Tip. The needle was orientated into the desired position using a micromanipulator (World Precision Instruments) and a stereomicroscope (Nikon, SMZ 745). Fine tweezers were then used to break the needle tip, and the inoculum drop was then calibrated to 1 nl using a graticule (Pyser Optics) coated with mineral oil. The 18th-20th dorsal somite of day 3 zebrafish larvae was then injected, and the infection site was timelapsed from 30 months post infection (mpi).

#### Microscopy

Larvae were mounted in a 1% low-melting-point agarose solution (Affymetrix, 32,830) containing 0.168 mg/ml tricaine. Agarose was covered with 500 μl clear E3 solution. Imaging was performed from 30 min until 3 h post injury using a 40× oil objective [UplanSApo 40× oil (1.3 NA)] on an UltraVIEWVoX spinning disk confocal laser imaging system (Perkin Elmer) with a Hamamatsu C9100-50 EM-CCD camera. Fluorescence for eGFP and pHrodo™ Green was acquired using an excitation wavelength of 488 nm; emission was detected at 510 nm. Fluorescence for pHrodo™ Red and Crimson was acquired using 525 nm emission and detected at 640 nm. 25-40 µm *z*-stacks were captured with a step interval of 1.5 µm.

#### Image analysis

Images were processed using Fiji and MATLAB^®^ (MathWorks). Phagosomes were analysed for a mean of 72 min (6.4-191.7±56.7 min). Phagosomes were tracked using TrackMate (Ershov et al., 2022). On average, phagosomes were analysed for 72 min (6.4-191.7±56.7 min). The position of the phagosomes was obtained using the tracking plug-in TrackMate (Tinevez et al., 2017), and all subsequent analysis was performed in MATLAB. The volumetric analysis of the phagosomes was performed with an intensity based-segmentation on each of the 3D volumes. All channels were smoothed in 3D with a box kernel of size 3×3×3 voxels, and then intensity levels were used to perform an intensity-based segmentation. For the green channel, two intensity levels were employed – one low that corresponded to the whole neutrophil and one high that corresponded to the phagosome. The red channel only required one level that corresponded to the bacteria. The volumes of the bacteria inside and outside the neutrophil were measured, and the ratio of inside and outside was calculated, as well as the average intensities for all frames. To display the volumes, isosurfaces were calculated and displayed as meshes (Fig. 3A). To calculate the intensities of the phagosomes over time, the positions were recorded as previously mentioned, and then the intensities of all pixels within a ring of radius of 6 pixels were extracted. The average intensity inside the ring was calculated, as well as the intensities as a function of the angle; in particular, 21 directions were calculated to visualise whether the intensity was uniform around the ring or higher on one side than on the other. For this study, nine phagosomes in four different datasets were analysed. Each track consisted of a 2D dataset of dimensions (number of time frames×21, where ‘21’ corresponded to the angles of the ring). The intensity values were low-pass filtered to smooth the results (Fig. 1A). Finally, the intensity values of the nine tracks were averaged to calculate mean and s.d. First, intensities were normalised by subtracting the minimum value of the track and then dividing by the maximum so that the range of values was within 0 and 1. Next, the tracks were shifted in time so that all the start of all tracks coincided in a new time 0 and could be compared. Then mean and s.d. per time frame were calculated (Fig. 1A). The code is publicly available at GitHub: https://github.com/reyesaldasoro/RingFluorescence.

Image analysis of human neutrophils was performed using Harmony software (Revvity) by segmenting pHrodo™ Red-stained bacteria inside neutrophils and then measuring the mean intensity of Lucifer Yellow within the mask. Threshold was applied to determine the Lucifer Yellow-positive and -negative fraction of bacteria-filled phagosomes.

#### Preparation of beads

50 µl of a 2% solution of 1 µm crimson beads (Thermo Fisher Scientific, F8816) was diluted, washed and then re-suspended in 500 µl PBS. 1 nl of this suspension was then injected into the 18th-20th dorsal somite of day 3 zebrafish larvae.

#### Preparation of neutrophil actin reporter

*Tg(mpx:Lifeact-Ruby)sh608* was made by injecting single-cell larvae with the destination plasmid, *Tol2-mpx:Lifeact-Ruby* (a gift from Anna Huttenlocher, University of Wisconsin, Madison, USA) with the *Tol2* transposase RNA generated as previously described (Prajsnar et al., 2012).

#### Injection with CellROX™ reagent

1 µl CellROX™ Deep Red Reagent was added to 50 µl of 1500 cfu/nl *S. aureus* culture.

#### Preparation of human neutrophils

Human neutrophils were isolated by Plasma-Percoll density gradient centrifugation from whole blood of healthy donors as previously described (Pertoft et al., 1978). The study was carried out with written informed consent obtained from each donor. Ethical approval was obtained from the Edinburgh Medical School Research Ethics Committee (21-EMREC-41). Freshly isolated neutrophils were re-suspended at 0.3×10^6^ cells/ml in Phenol Red-free Roswell Park Memorial Institute (RPMI) 1640 medium (Thermo Fisher Scientific), supplemented with 10% (w/v) heat-inactivated fetal bovine serum (FBS; PromoCell) and 25 mM HEPES buffer solution (Sigma-Aldrich). Neutrophils were cultured in a 96-well #1.5, ibiTreat polymer-treated plate (ibidi, 89606). Freshly isolated neutrophils (9×10^4^ cells) were incubated at 37°C for 30 min. pHrodo™ Red-stained *S. aureus* (JE2) was then added, and the plate was spun at 100 ***g*** for 1 min to synchronise phagocytosis before incubating for 15 min at 37°C. Neutrophils were then stained with 10 µg/ml CellMask™ Deep Red plasma membrane stain (Invitrogen, C10046) before incubating for another 10 min at 37°C. Neutrophils were then timelapsed for 5 min before the addition of 50 µg/ml Lucifer Yellow (Thermo Fisher Scientific, L453). A 60 min timelapse of the same neutrophils commenced immediately after the addition of Lucifer Yellow.

#### Microscopy of human neutrophils

Live imaging of human neutrophils was performed on an Opera Phenix (Perkin Elmer), using excitation wavelengths of 425, 561 and 647 nm. Neutrophils were maintained at 37°C using the in-built incubator of the Opera Phenix.

#### Drug treatment of zebrafish

Day 3 zebrafish larvae were immersed in 1 µm AS605240 and 100 µm CAL-101 (Selleckchem) in 1% dimethyl sulfoxide (DMSO) in E3 medium. Larvae were incubated in inhibitors at 28°C for 30 min before imaging. Controls were similarly immersed in 1% DMSO in E3 medium.

#### Diphenyleneiodonium (DPI) inhibition and ROS imaging within *S. aureus*-infected zebrafish neutrophils

DMSO- and DPI-treated (100 µm) neutrophil-repleted (*irf8* knockdown) (Salamaga et al., 2017) ABTL larvae at 48 h post fertilisation (hpf) were co-injected with 1500 cfu of pHrodo™ Red-labelled JE2 and 10 mM 2′,7′-dichlorofluorescin diacetate (Sigma-Aldrich, 287810). At 1 h post infection, larvae were imaged using a Zeiss LSM 900 with a 20× objective (0.8 NA). The 488 and 561 nm laser lines were used. pHrodo™ Red and dichlorofluorescein (DCF) fluorescence intensity was quantified using an ImageJ custom script called Fish Analysis v5 (Salamaga et al., 2017). All bacterial clusters were identified based on their pHrodo™ Red fluorescence, and non-phagocytosed bacteria were disregarded. The fluorescence intensity of the DCF surrounding the phagocytosed bacteria (0.5 µm radius) was quantified. Subsequently, we calculated the ratio of the DCF fluorescence surrounding the bacteria to the signal from non-infected phagocytes to normalise the results to the baseline ROS signal in neutrophils of both DMSO and DPI groups.

#### Zebrafish survival curves

1900 cfu of pHrodo™ Red-labelled and unstained JE2 were injected into the circulation of 48 hpf ATBL larvae as described previously (Salamaga et al., 2017).

#### Quantification and statistical analysis

Prism software (GraphPad Software) was used to perform statistical analysis. Precision measures, *n* values and statistical tests used are indicated in figure legends. *P*≤0.05 was considered significant.

## Supplementary Material

10.1242/dmm.052042_sup1 Supplementary information
