# Correction: A subset of neutrophil phagosomes is characterised by pulses of Class I PI3K activity

**DOI:** 10.1242/dmm.052685

**Published:** 2025-10-22

**Authors:** Clare F. Muir, Constantino Carlos Reyes-Aldasoro, Tomasz K. Prajsnar, Bartosz J. Michno, Justyna Cholewa-Waclaw, Yin X. Ho, Audrey Bernut, Catherine A. Loynes, Stone Elworthy, Kieran A. Bowden, Ashley J. Cadby, Lynne R. Prince, Jason S. King, Felix Ellett, Alison M. Condliffe, Stephen A. Renshaw

There were errors in *Dis. Model. Mech.* (2025) **18**, dmm052042 (doi:10.1242/dmm.052042).

There were numerous mismatches in the references and citations that have now been corrected in the XML and PDF versions of the article.

We apologise to authors and readers for these errors and any inconvenience they may have caused.

