# Supplementary material for: A subset of neutrophil phagosomes is characterised by pulses of Class I PI3K activity
Source: Dis Model Mech. 2025 Aug 29;18(9):dmm052042. doi: 10.1242/dmm.052042 (PMC12444860; doi:10.1242/dmm.052042)
Supplement: Supplementary information [file dmm-18-052042-s1.pdf]

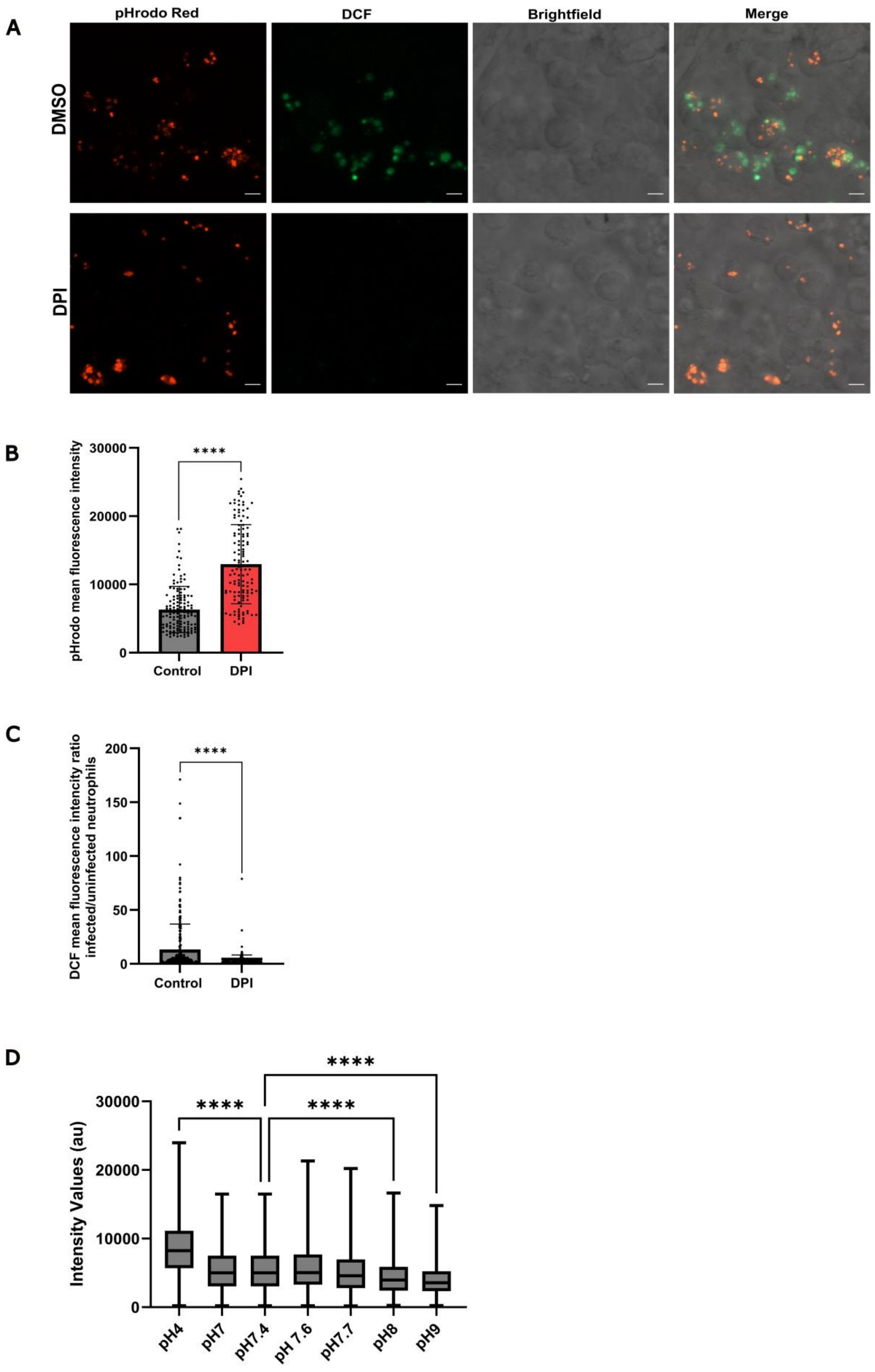

Disease Models & Mechanisms • Supplementary information

Fig. S1. NADPH oxidase drives the alkalinisation of the neutrophil phagosome. (A) Confocal images illustrating that the fluorescence of pHrodo Red-labelled internalised bacteria is higher and that the fluorescence of the ROS detector, DCF, dims when NADPH oxidase is inhibited in zebrafish larvae. **(B)** Quantification of the fluorescence of pHrodoRed stained *S.aureus* in control (DMSO) and NADPH oxidase-inhibited (DPI) zebrafish larvae, 11 larvae analysed from 2 independent experiments. Number of internalised bacterial clusters analysed: DMSO – 281, DPI – 251. **(C)** Quantification of the fluorescence of the ROS detector, DCF, in control (DMSO) and NADPH oxidase inhibited (DPI) zebrafish larvae, 11 larvae analysed from 2 independent experiments. Number of internalised bacterial clusters analysed: DMSO: 281, DPI: 251. **(D)** Quantification of the fluorescence of pHrodo Red stained *S.aureus* in different pH PBS solutions.

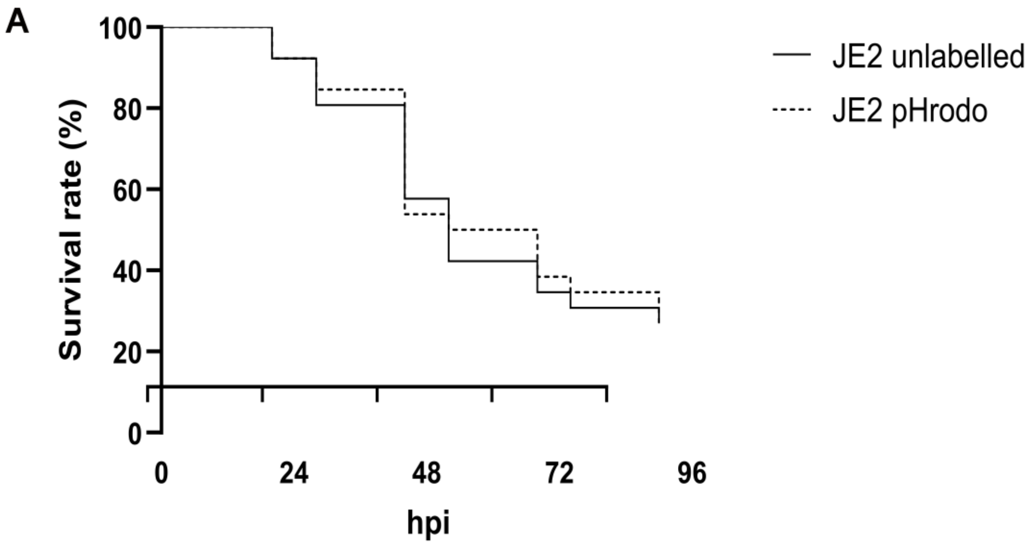

Fig. S2. (A) pHrodo staining does not alter the pathogenicity of *S.aureus*. 52 larvae analysed from 1 experiment.

Disease Models & Mechanisms • Supplementary information

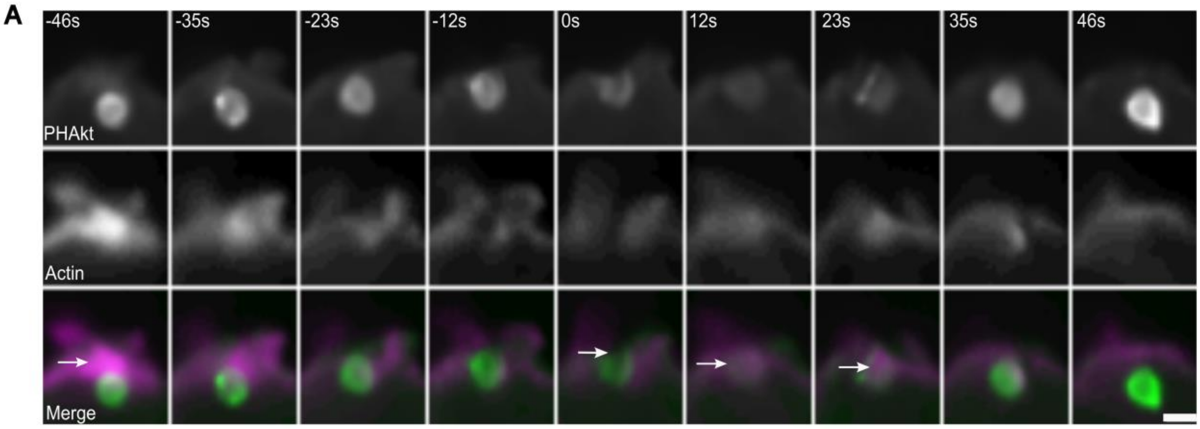

Fig. S3. Sequential images capturing the dynamics of PHAkt-eGFP and actin when bacteria are released and recaptured from a phagosome. Cortical actin fibres are visualised overlying the phagosome at 46s (arrow). At 0s, arrow shows the separation of cortical actin filaments as pulsing phagosome reopens onto the neutrophil surface. At 23s, actin filaments shape the phagocytic cup and surround the phagosome (arrow). Scale Bar = 2µm.

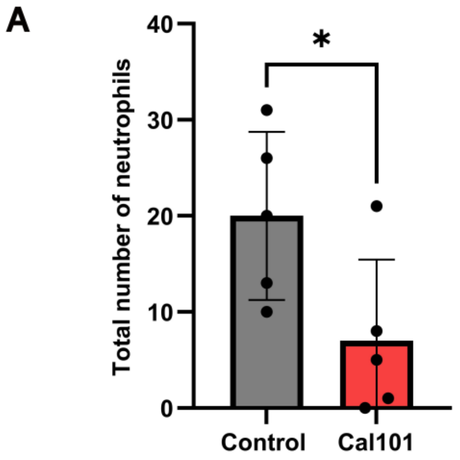

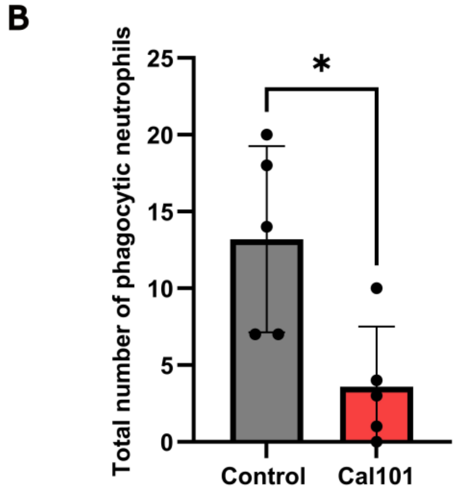

Fig. S4. CAL-101 reduces the number of neutrophils recruited to an *S.aureus* infection site. **(A)** Quantification of the number of neutrophils at an *S.aureus* infection site 2 hours post-infection following 30mins incubation with 100µm of CAL-101. Data shown is the mean +/- SD from 5 independent larvae, 5 experiments. **(B)** Quantification of the number of phagocytic neutrophils at a *S.aureus* infection site 2 hours post-infection following 30mins incubation with 100µm of CAL-101. Data shown is the mean +/- SD from 5 independent larvae, 5 experiments.

Disease Models & Mechanisms • Supplementary information

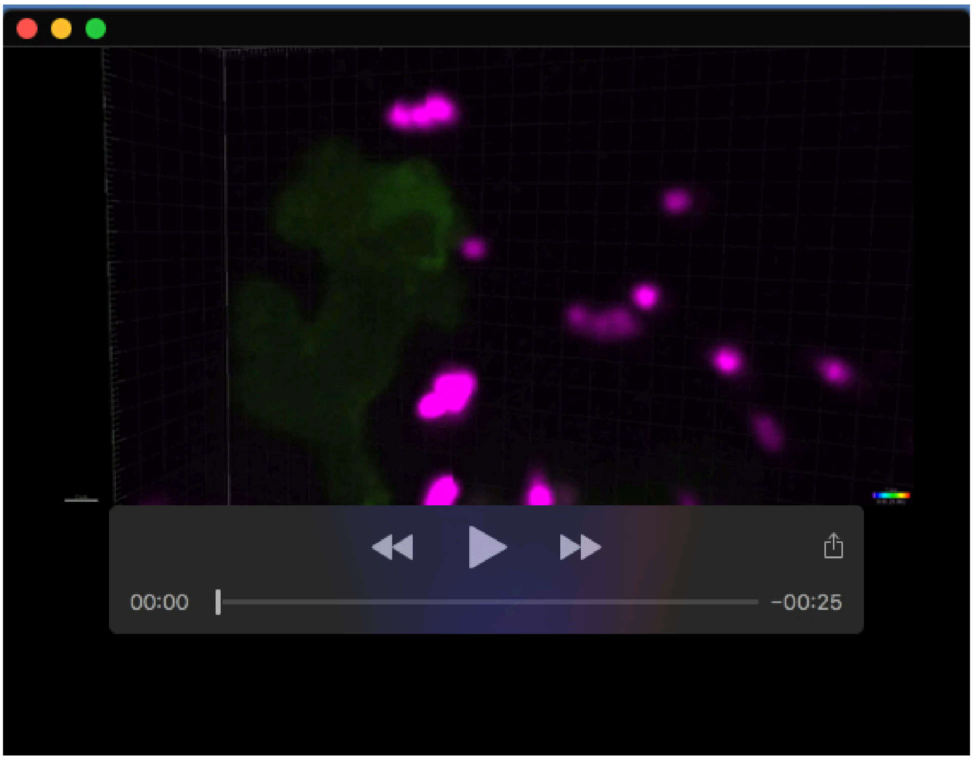

Movie 1. Dynamics of PHAkt-eGFP on neutrophil phagosomes following infection with *S.aureus*. Minimal PHAkt-eGFP recruits during early formation of the phagocytic cup. PHAkt-eGFP recruits strongly at sites of cup closure (white arrows) and uniformly to the phagosome membrane after cup formation (white arrows). Green = PHAkt-eGFP. Magenta = pHrodo™ Red stained *S.aureus*.

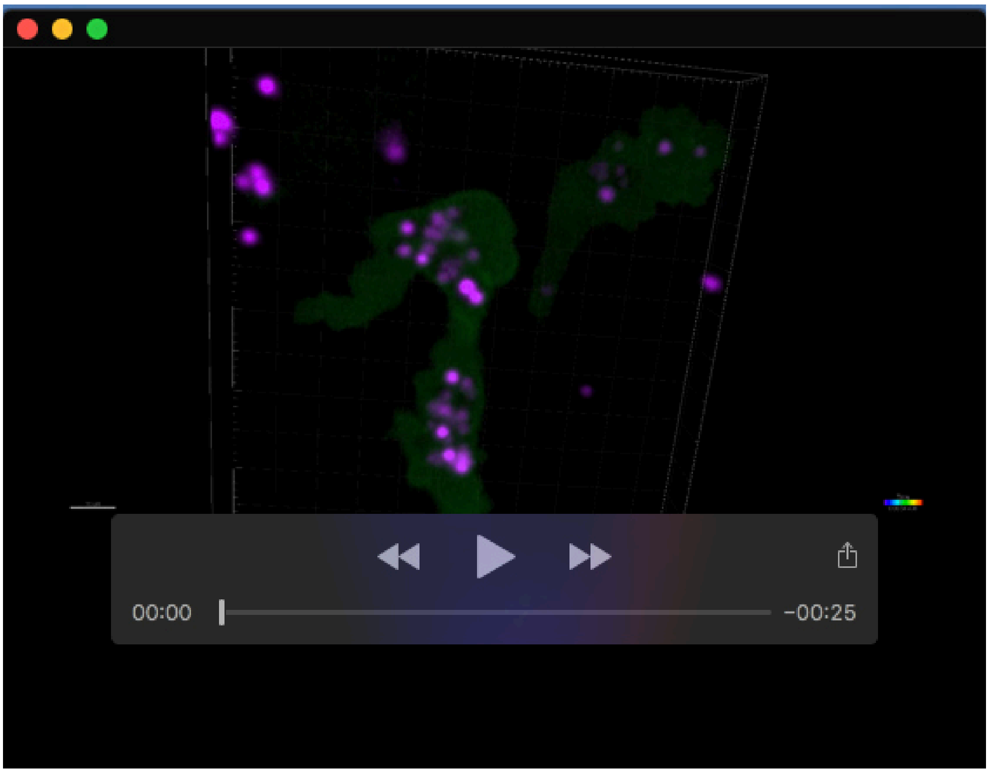

Movie 2. Pulsatile recruitment of PHAkt-eGFP to a neutrophil phagosome containing *S.aureus*. Green = PHAkt-eGFP. Magenta = pHrodo™ Red stained *S.aureus*.

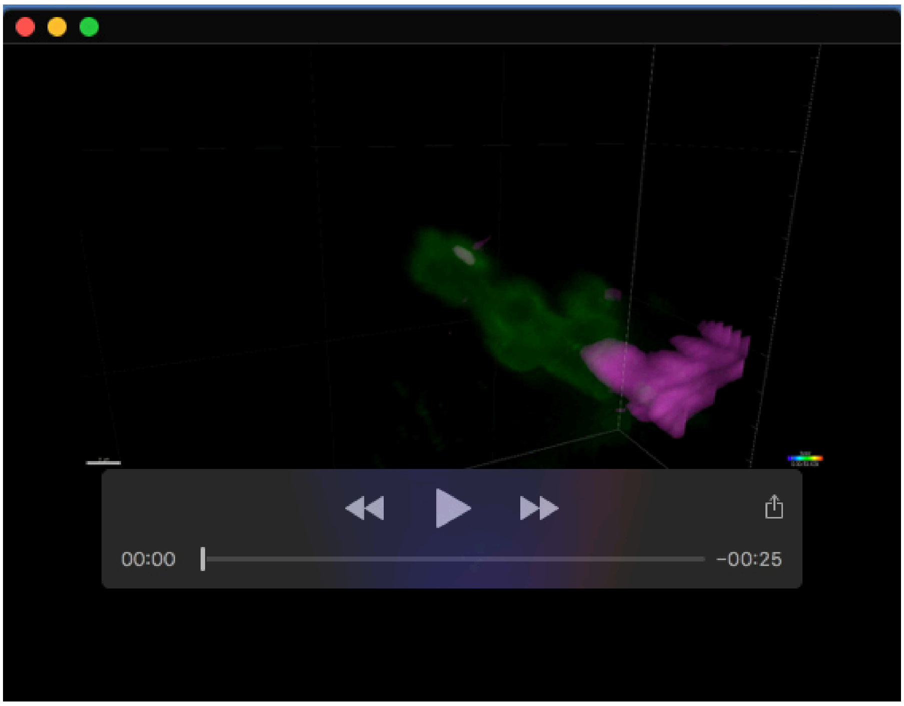

Movie 3. Pulsatile recruitment of PHAkt-eGFP to a neutrophil phagosome containing *Mycobacterium abscessus*. Green = PHAkt-eGFP. Magenta = pHrodo™ Red stained *M.abscessus*.

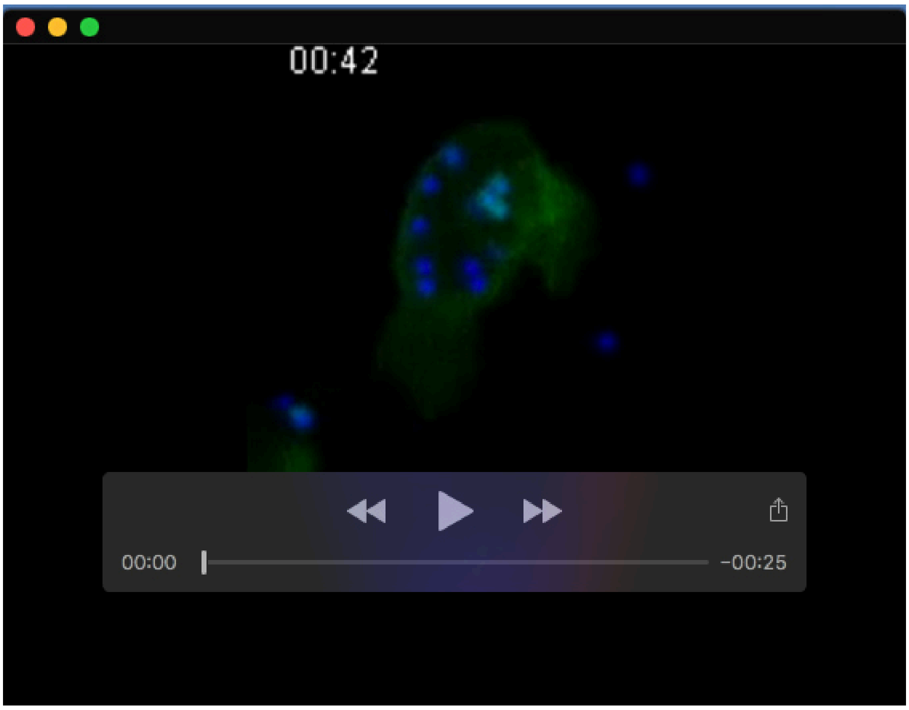

Movie 4. Pulsatile recruitment of PHAkt-eGFP to a neutrophil phagosome containing 1µm latex beads (blue spheres). 1µm bead is within the neutrophil at the start of the movie. PHAkt-eGFP recruits to the phagosome membrane (3m 5s) (Pulse). PHAkt-eGFP then dissipates from the phagosome membrane (8m 34). Green = PHAkt-eGFP. 1µm latex beads (blue spheres).

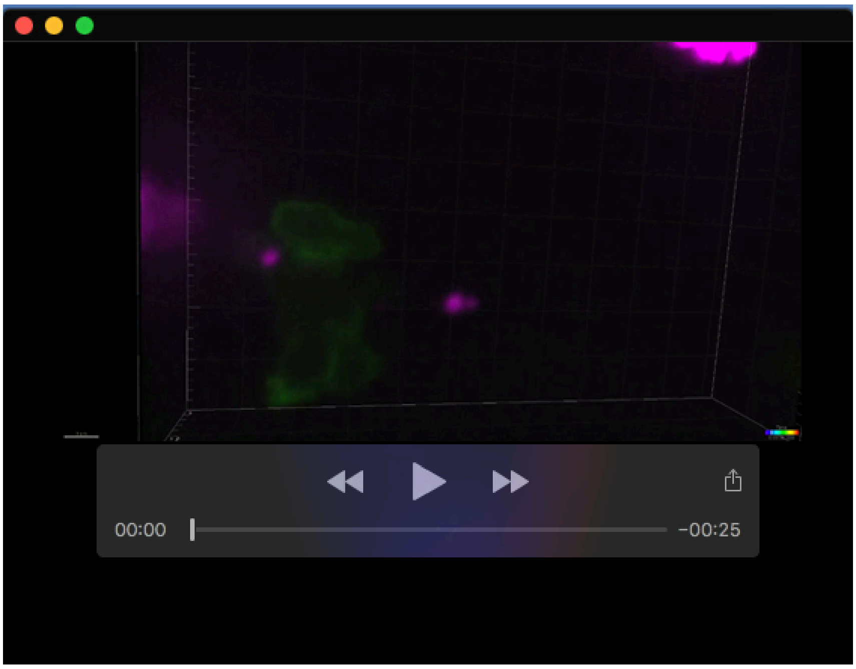

Movie 5. Neutrophil expelling and recapturing *S.aureus*. A neutrophil phagocytoses *S.aureus* (12s). PHAkt-eGFP (green) identifies the plasma and phagosome membranes. *S.aureus* is expelled from the phagosome onto the surface of the neutrophil (29s). *S.aureus* is recaptured by the neutrophil (1st pulse) (33s). Green = PHAkt-eGFP. Magenta = pHrodo™ red stained *S.aureus*.

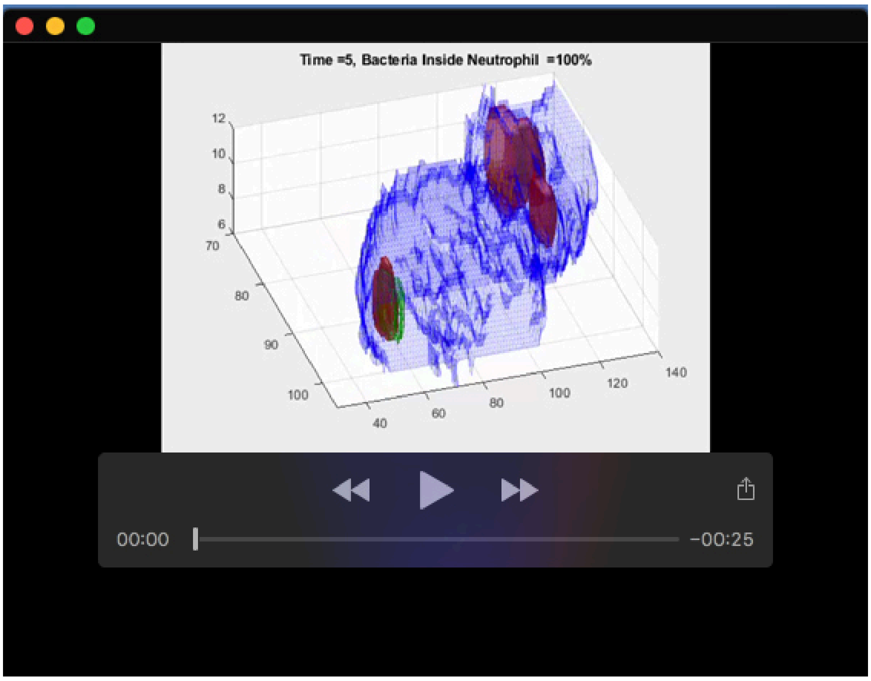

Movie 6. 3D reconstruction of a pulsing phagosome showing that a neutrophil phagosome reopens and recloses. *S.aureus* is within a phagosome at the start of the movie. PHAkt-eGFP recruits to the phagosome. PHAkt-eGFP diminishes from the phagosome membrane and *S.aureus* is released from the phagosome onto the surface of the neutrophil. *S.aureus* is recaptured by the neutrophil and PHAkt-eGFP re-recruits to the phagosome membrane (1st pulse). Yellow cage shows the % of the bacteria which is expelled from the phagosome. Green = PHAkt-eGFP. Red = pHrodo™ red stained *S.aureus*.

Disease Models & Mechanisms • Supplementary information

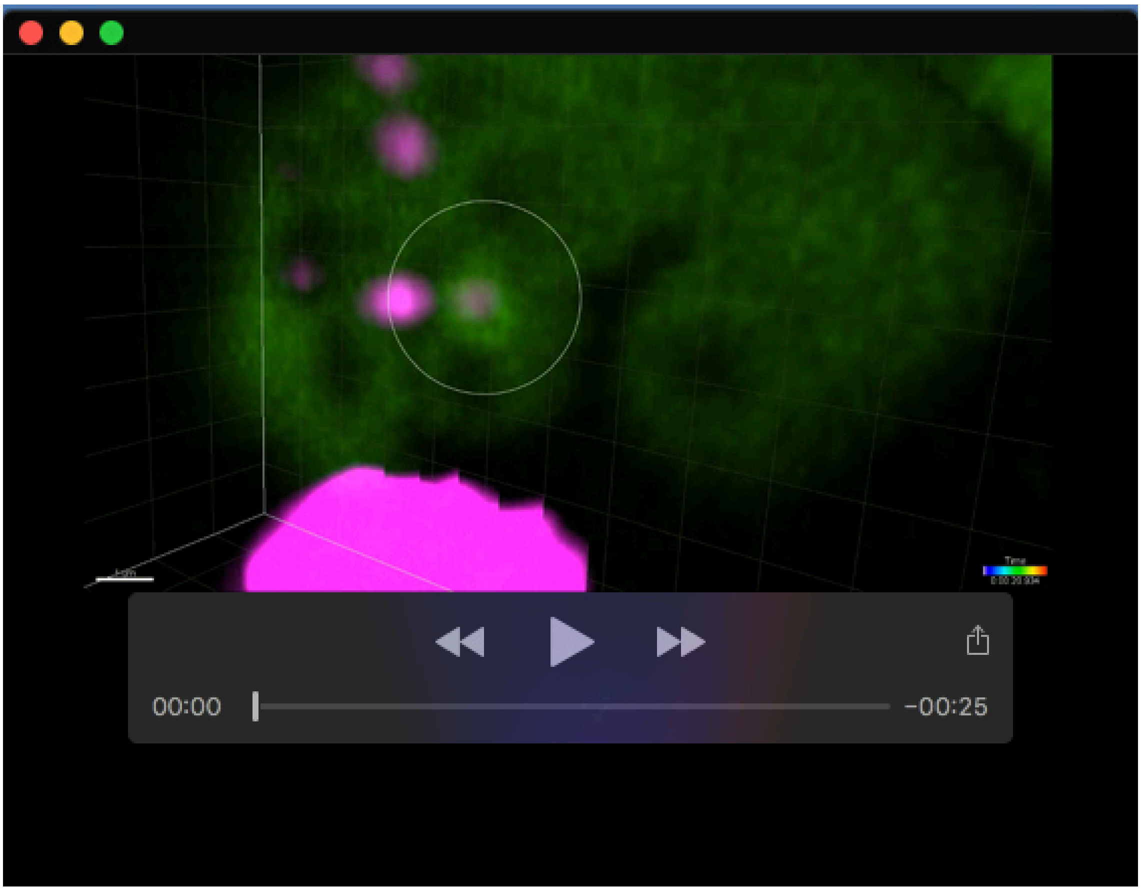

Movie 7. PI3Kγ inhibition prevents pulsatile recruitment of PHAkt-eGFP to neutrophil phagosomes. *S.aureus* is within a phagosome at the start of the movie (white circle). *S.aureus* is then released from the phagosome (28s). Neutrophil then attempts to rephagocytose *S.aureus* (1m 43s) (blue dot) but PHAkt-eGFP does not re-recruit (pulse) to the phagosome. Green = PHAkt-eGFP. Magenta = pHrodo™ Red stained *S.aureus*.
